# Construct validity of the Actiwatch‐2 for assessing movement in people with profound intellectual and multiple disabilities

**DOI:** 10.1111/jar.12789

**Published:** 2020-07-29

**Authors:** Helena J. M. van Alphen, Aly Waninge, Alexander E. M. G. Minnaert, Wendy J. Post, Annette A. J. van der Putten

**Affiliations:** ^1^ Department of Inclusive and Special Needs Education University of Groningen Groningen the Netherlands; ^2^ Research Group Healthy Ageing, Health Care and Nursing Hanze University of Applied Sciences Groningen the Netherlands

**Keywords:** outcome assessment, physical activity, profound intellectual and multiple disabilities, psychometric properties

## Abstract

**Background:**

Valid measures to assess either small or assisted performed movements of people with profound intellectual and multiple disabilities (PIMD) are required. We analysed the construct validity of the Actiwatch‐2 to assess movement in people with PIMD.

**Method:**

Twenty‐two persons with PIMD were video recorded while wearing an Actiwatch‐2. We used 15s‐partial‐interval recording to record upper body movement, body position and activity situation. Multilevel analyses were used to evaluate if the Actiwatch‐2, based on produced counts, could detect changes in these factors.

**Results:**

The presence versus absence of upper body movement and an activity situation in which participants were involved versus not involved resulted in significantly higher counts, with a large variety in predicted counts between participants. No relationship between body position and counts was found.

**Conclusions:**

The Actiwatch‐2 seems able to assess obvious upper body movement in people with PIMD, and whether there is involvement in an activity situation.

## 1 INTRODUCTION

A wide variety of movement activities are used in current practice to activate people with profound intellectual and multiple disabilities (PIMD) (Van Alphen, Waninge, Minnaert, & Van der Putten, 2019). These movement activities require a special approach regarding the attitude towards people with PIMD, because of their limitations in cognitive‐ and motor functioning (Nakken & Vlaskamp, 2007). People with PIMD are fully wheelchair dependent or require personal assistance to mobilize and change body position (Nakken & Vlaskamp, 2007). Technical devices and extensive support are needed to accommodate people with PIMD and supporting even very small movements of the limbs and postural changes of people with PIMD. In current practice, demanding activities, such as bouncing on a bouncy castle (Van der Putten, Houwen, & Vlaskamp, 2014), activities in a swimming pool (e.g. Watsu: Dull, 2004), and power‐assisted exercises using machines that assist people with PIMD to passively move their arms and legs are used (Bossink, Van der Putten, Waninge, & Vlaskamp, 2017). In addition, small‐scale activities are integrated in the daily support, such as activation to lift of an arm, standing using a standing tool and assist to turn over during dressing (Lancioni et al., 2005; Van Alphen et al., 2019; Van der Putten, Vlaskamp, Reynders, & Nakken, 2005). In addition, recently, also new technologies such as an interactive ball are introduced to increase body movement in persons with PIMD (Embregts et al., 2020; Van Delden et al., 2020).

These movement activities can be used for a wide range of goals and encourage different domains of human functioning, such as the motor domain, but also beyond the motor domain, for example in social and cognitive functioning (Embregts et al., 2020; Houwen, Van der Putten, & Vlaskamp, 2014; Jones et al., 2007; Van Alphen et al., 2019; Van der Putten et al., 2014). As a result, several studies recommend movement activities to be directed towards individual and specific measurable goals integrated within the overall support provided for people with PIMD (Bossink et al., 2017; Van Alphen et al., 2019; Van der Putten et al., 2005; Wessels, Bossink, & van der Putten, 2017). To identify whether goals are achieved and to what extent improvement of movement has contributed to outcomes on different domains, researchers and practitioners should be able to accurately assess the amount of movement of people with PIMD.

In general, movement (or physical activity) is assessed based on energy expenditure or the execution of movements in daily life (e.g. steps per day: Hilgenkamp, Reis, Van Wijck, & Evenhuis, 2012). Several studies have been performed into the validation of a wide range of devices to assess movement in ambulatory people, but hardly in non‐ambulatory people such as people with PIMD (Berlin, Storti, & Brach, 2006; Warms & Belza, 2004). In addition, algorithms that predict the activity energy expenditure of people with PIMD are lacking (Waninge et al., 2013). Moreover, measurement evidence (e.g. validity and reliability) among subgroups of people with intellectual disability, such as people with PIMD, are lacking in this field (Pitchford, Dixon‐Ibarra, & Hauck, 2018). Therefore, there is an urgent need for research into instruments measuring movement in people with PIMD. Most movements of people with PIMD are either small and assisted or passively performed. Therefore, we suggest that instruments measuring movement in people with PIMD should capture actively as well as assisted and passively performed movements. In addition, even small movements of the limbs performed from different body postures (i.e. lying, sitting and standing) as well as changes in body position are important to identify in people with PIMD, because these are not self‐evident.

To date, a few subjective and objective measures are used to assess the movement behaviour of people with PIMD (Van Delden & Reidsma, 2018; Van der Putten, Bossink, Frans, Houwen, & Vlaskamp, 2017; Waninge et al., 2013). A previous study investigated the degree and type of strategies offered to facilitate movement in people with PIMD by the use of a diary (Van der Putten et al., 2017). This study did provide a valuable insight into the number of transfers, relocations and motor activities offered in the support of people with PIMD (Van der Putten et al., 2017). However, it did not focus on the actual amount of movement of people with PIMD. In addition, diaries in general are susceptible to inaccurate recall and in comparison with objective measures less accurate to assess the amount of movement performed. Objective measures such as heart rate monitors have been used to provide an insight into the daily activity patterns of persons with PIMD (Waninge et al., 2013). Heart rate monitors maybe useful to roughly evaluate initiatives directed at the facilitation of movement, but it is unclear if those monitors based on heart rate patterns also could identify passively and assisted performed movements of people with PIMD. In addition, heart rate patterns are influenced by differences in physiological responses and with time of day, age, and probably also other personal and psychosocial factors (Waninge et al., 2013; Warms, 2006). As a result, the influence of movement on heart rate in people with PIMD is not fully clear. Automatic measurements of movement based on video recordings have also been used in people with PIMD (Van Delden & Reidsma, 2018). In the simplified motion energy analysis, for instance, the amount of pixels that changed beyond a certain threshold is measured. Although, the use of persuasive technological measurements is highly valued, the outcomes can become difficult due to unforeseen side‐effects and incorrect values (e.g. influence of auto‐focus, shaking camera, moving material and other persons who entered the view of the camera) (Van Delden & Reidsma, 2018). All in all, movement can be measured in different ways, but specific instruments with clear psychometric properties are needed to assess the amount of movement of people with PIMD.

Accelerometers can provide objective and continuous information about the duration, frequency and intensity of movements and are relatively easy to wear (Ainsworth, Cahalin, Buman, & Ross, 2015; Berlin et al., 2006). An Actiwatch, a wrist‐worn accelerometer, is originally developed to measure rest‐activity patterns based on body movement and is previously used in the support of people with PIMD to investigate sleep problems (Drenth, Poppes, & Vlaskamp, 2007; Van de Wouw, Evenhuis, & Echteld, 2013; Van Dijk, Hilgenkamp, Evenhuis, & Echteld, 2012). Because an Actiwatch records wrist accelerations which are directly related to the amount of movement performed, this instrument may be useful to distinct between facilitated movements and small involuntary movements in people with PIMD. In addition, an Actiwatch may be able to distinct activities performed from different body postures and possibly also different activity situations and ways of stimulation. Moreover, actively performed as well as passively and assisted performed movements will be identified by an Actiwatch which is important particularly in people with PIMD because of their severe motor disabilities.

Only a few studies have investigated (in other populations than people with PIMD) whether the Actiwatch‐2 (Philips, Respironics) can be used as a measure of movement behaviour (Lambiase, Gabriel, Chang, Kuller, & Matthews, 2014; Lee & Suen, 2017; Neil‐Sztramko, Rafn, Gotay, & Campbell, 2017). These studies suggest that an Actiwatch‐2 is able to discriminate different intensities of movement activity (Neil‐Sztramko et al., 2017), although may better capture low‐intensity activities instead of higher intensity activities (Lambiase et al., 2014; Warms, 2006). This may be particularly pertinent for people with PIMD. In addition, an instrument such as an Actiwatch‐2 that could measure both sleep and movement behaviour simultaneously will reduce the burden on participants with PIMD. Moreover, the Actiwatch‐2 is already used to measure sleep of persons with PIMD on a regular basis. Hence, the research could benefit from the fact that participants as well as their support professionals are already acquainted with the use of this instrument.

The validity of the Actiwatch‐2 to assess movement in people with PIMD has not been previously investigated. The purpose of this study was to investigate the construct validity of the Actiwatch‐2 to assess movement in people with PIMD. We evaluated if the Actiwatch‐2 could detect observed changes in upper body movement, body position and activity situation. We have added activity situation to this study, because movement in persons with PIMD largely depend on stimulation by the environment and could result from different activity situations and ways of stimulation, even when not directly aimed at movement. Future research on the effectiveness of movement interventions may benefit from the results if for instance passive and active participation in movement can be distinguished.

## 2 METHOD

### 2.1 Participants

In the present study, the participants were enrolled in an intervention study registered at the Netherlands Trial Register (number 6627), which was approved by the Ethics Committee for Pedagogical Sciences and Educational Science of the University of Groningen. Based on funding cooperating parties, participants were recruited by physical therapists of three different residential facilities offering 24‐hr support to people with intellectual and visual disabilities, including people with PIMD. For 26 participants, written informed consent was obtained from parents or legal representatives. Inclusion criteria were (a) severe or profound intellectual disability (intelligence quotient (IQ) under 35 points or a developmental age up to 36 months), (b) severe or profound motor disability (classified as Gross Motor Function Classification System (GMFCS) IV or V: Palisano et al., 2000), and (c) a continuous need for support for all activities in daily life (Nakken & Vlaskamp, 2007; WHO, 2001). In addition, all participants had moderate to profound visual impairment or blindness (a visual acuity of less than 0.3 (WHO, 2016)), because they were recruited from the cooperating residential facility for people with visual impairment and intellectual disability. Participants of the above‐mentioned study were included in the present study if they had available Actiwatch and video data collected within the same time frames. Three participants were excluded because of missing Actiwatch data due to oversensitivity or reluctance to wear the device on their wrist. In addition, one participant was excluded because of missing video data. Therefore, the current study is based on 22 participants with PIMD (11 males and 11 females) with a mean age of 35.1 ± 13.6 years. Table 1 shows the characteristics of the participants in terms of mobility and health problems.

**Table 1:** Participant characteristics in terms of mobility and health problems

Participant	Sex	Age (years)	Mobility	Health problems
1	Male	19	Requires heavy assistance to mobilize	Visual impairment, epilepsy
2	Female	48	Fully wheelchair dependent	Visual impairment, epilepsy
3	Female	50	Fully wheelchair dependent	Visual and auditory impairment
4	Female	61	Fully wheelchair dependent	Blindness and auditory impairment, epilepsy
5	Female	52	Fully wheelchair dependent	Visual impairment, epilepsy
6	Male	45	Fully wheelchair dependent	Blindness, epilepsy
7	Male	46	Fully wheelchair dependent	Visual and auditory impairment, epilepsy
8	Male	39	Fully wheelchair dependent	Deaf blindness
9	Male	36	Fully wheelchair dependent	Visual and auditory impairment, epilepsy
10	Male	24	Fully wheelchair dependent	Blindness and auditory impairment, epilepsy
11	Male	37	Fully wheelchair dependent	Visual impairment, epilepsy
12	Male	11	Fully wheelchair dependent	Blindness
13	Female	31	Fully wheelchair dependent	Blindness, epilepsy
14	Male	31	Fully wheelchair dependent	Blindness, epilepsy
15	Male	25	Fully wheelchair dependent	Blindness
16	Female	30	Fully wheelchair dependent	Blindness, epilepsy
17	Female	27	Fully wheelchair dependent	Blindness, epilepsy
18	Male	41	Fully wheelchair dependent	Blindness and auditory impairment, epilepsy
19	Female	23	Fully wheelchair dependent	Visual impairment, epilepsy
20	Female	26	Fully wheelchair dependent	Blindness and auditory impairment, epilepsy
21	Female	17	Fully wheelchair dependent	Blindness and auditory impairment, epilepsy
22	Female	56	Requires heavy assistance to mobilize	Visual and auditory impairment

### 2.2 Procedures

The data were retrieved from the above‐mentioned intervention study including three measurement periods that lasted two weeks each measurement period. Per measurement period, wrist accelerations of the dominant wrist (their most mobile arm/hand) were measured with an Actiwatch‐2 for at least seven consecutive days, 24 hr per day. In addition, participants were video recorded during their regular program (without prescription of any activity by the researchers and except for caring activities where clothes were taken off) each measurement period eight times for about 15 min. The participants were video recorded in the morning (four times) and in the afternoon (four times) spread over different weekdays. The video recordings have captured at least the entire upper body (from the waist) of the participants. The video recordings were made by a tripod, but the camera was moved by hand on the tripod when the participant (was) moved through the room. Each video recording contained a first frame with a sheet indicating the time of the day for validation of the time frames to be used. The data from similar periods of time were used for the analysis and determined based on the manually set time of the cameras equated to the automatically set time of the Actiwatch.

### 2.3 Measurements

#### 2.3.1 Actiwatch measurements

Actiwatch‐2 (Philips, Respironics) data were collected with an epoch duration of 15 s. The Actiwatch‐2 contains an acceleration‐responsive piezoelectric sensor and is set up to record the intensity, frequency and duration of movements which is converted into voltage. This means that an increase in speed and motion produces an increase in voltage (sampling rate 32 Hz), which was integrated and stored as an activity count in the Actiwatch memory reflecting the peak acceleration per 15 s. Actiwatch data were transferred offline to a computer and automatically stored in activity counts by date and time using the Philips Actiware 6.0.9. software.

#### 2.3.2 Video‐based observations

Partial interval coding (Cooper, Heron, & Heward, 2007), every 15 s of each video recording, was used for coding the occurrence of upper body movement, body position and activity situation. The occurrence of upper body movement was scored as present for obvious trunk movements (rotation, flexion or extension of the vertebral column) and movements of the arms (elbow flexion and extension, shoulder external rotation, abduction and adduction). These movements could be performed actively or with assistance of technical devices or support. The occurrence of upper body movement was scored as absent if none or very small movements occurred (e.g. pronation and supination of the forearms and hands, small involuntary vibrating movements, and minimal shifting of the arms and hands). Body position (adapted from Kozey‐Keadle, Libertine, Lyden, Staudenmayer, & Freedson, 2011) was coded in four categories as presented below:

1. Lying: Participants were in a horizontal position, parallel to the ground.
2. Sitting: Participants had some of their body weight supported by the buttocks or thighs. The upper body was not parallel to the ground.
3. Standing still: Participants were upright and standing still.
4. Standing/moving: Participants were engaged in walking activity with physical assistance or use of a body support walker, for example.

Activity situation was coded in four categories indicating a different involvement of people with PIMD due to a different aim of stimulation in relation to movement activity (adapted from Special Heroes, 2013). The four categories are as follows:

1. Being present: Participants were present, but not actively engaged or involved in the activity situation. For example, audio‐visual activities, activities focusing on other participants in the same environment, or even no activities were provided and resulting in, for instance, movements arising from behavioural states.
2. Being part of: Participants were part of the activity situation, but not directly stimulated by their environment to move actively. For example, activities like massage, grooming or moments of social interaction were offered.
3. Passive participation: Participants were involved in activities with the help of support aimed at an active an engaged movement experience. For instance, the limbs of the participants were moved by powered exercise machines or participants experienced the wind while swinging and being moved in a hammock.
4. Active participation: Participants were actively involved and engaged in the activity situation and had a motorically active participation with little support. For instance, participants were eating independently (e.g. holding a cup or picking up a piece of bread), splashing the water while swimming, initiated bouncing movements on a bouncy castle, or were walking with physical assistance or use of a body support walker when positioned.

#### 2.3.3 Reliability

To ensure reliable coding, 12 video recordings (two randomly chosen video recordings of six participants) were coded by two independent observers. The interrater‐reliability was calculated by using Cohen's kappa (Cohen, 1960). The reliability was adequate: for coding the four body positions 100.0% agreement was reached (Cohen's kappa: 1.0). For coding the occurrence of movement as absent or present, the exact agreement was 87.1% (Cohen's kappa: 0.7). To ensure an optimal reliability for final coding, disagreements were discussed and resolved based on establishment of a more specified definition of movement and agreements on coding for missing data. A missing value was only coded when the participant was not visible for the full 15 s; otherwise, the highest‐rated category based on observation was given for the body position, activity situation and occurrence of movement. For example, if absence as well as presence of movement were seen during the 15 s, it was scored as the presence of movement. This was chosen because the Actiwatch data were collected with an epoch duration of 15 s.

### 2.4 Statistical analysis

To determine the construct validity of the Actiwatch‐2, it was analysed if the Actiwatch‐2 could detect changes in the occurrence of upper body movement (absence vs. presence), body position (lying, sitting, standing, or standing/moving), and activity situation (being present, being part of, passive participation or active participation) as scored based on observation. First, descriptive statistics were computed with the use of SPSS Statistics 25.0. For each participant, the mean activity counts and standard deviations for the absence and presence of movement and for each of the body positions and activity situations were calculated. Second, the relationship between the counts and occurrence of movement (absence vs. presence) was analysed using multilevel analyses by MLwiN 3. Multilevel analyses were used to consider the variation between participants (level 3) as well as between video recordings (level 2) and video‐based observations (level 1) within the participants. The multilevel analyses started with the random effects multilevel model without explanatory variables (empty model) with counts as dependent variable. Next, we added the variable occurrence of movement (presence vs. absence) to the model (fixed effect). In addition, we tested the random slope model for the variable occurrence of movement. Subsequently, we added body position (lying, sitting, standing or standing/moving) and activity situation (being present, being part of, passive participation or active participation) as covariates to the model. Significance testing of model parameters was done as described in Snijders and Bosker (2012). Deviance tests were used for model comparison (Snijders & Bosker, 2012). Assumptions were checked by plotting the model residuals for the final model. In the case of violation of assumptions, a logistic model for binomial responses was conducted using "lower category counts" and "higher category counts." As the distribution of counts contained more than half of the counts (59.7%) within a count range between 0 and 10 (for the absence of movement even 85.9%), we tested a model with lower category counts containing count range 0–10 and higher category counts including all other count values.

## 3 RESULTS

### 3.1 Data from similar periods of time

The participants had available Actiwatch data for at least one video recording (of about 15 min) with a maximum of 11 video recordings. As we used 15s‐partial‐interval recording, each video recording consisted of about 60 video‐based observations. On average, participants had 232.5 valid video‐based observations (min = 62, max = 706) with corresponding activity counts. This resulted in a total of 8,243 valid observations (34.3 hr) and simultaneous activity counts.

### 3.2 Relationship between movement and activity counts

**Figure 1:**
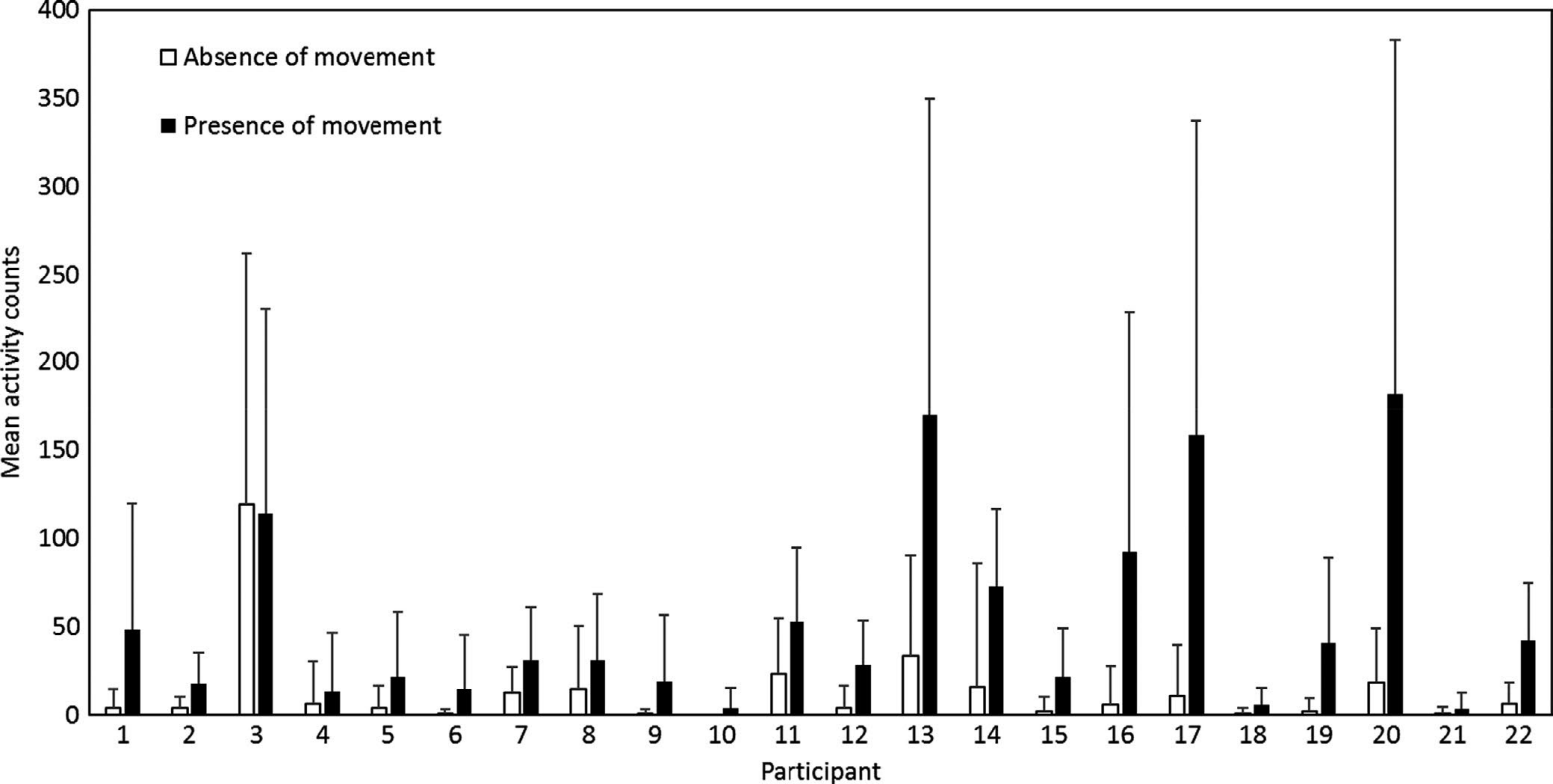
Mean activity counts (and standard deviation) per participant. Open bars: absence of movement. Filled bars: presence of movement

The mean number of counts was 12.5 times higher for the presence of movement (*M* = 90.1, *SD* = 139.3) compared with the absence of movement (*M* = 7.2, *SD* = 29.0) (See Table 2). As shown in Figure 1, there is a wide variety in the count range between participants, but the mean number of counts for all participants except one (participant 3) was higher for the presence of movement versus the absence of movement for each of the body positions and activity situations (see Figure 1 and Table 2). The results of the multilevel models are presented in Table 3. The mean number of counts of all participants was 38.7 varying with a standard deviation of 43.0 between participants (level‐3 variance = 1,850.2) (see Model 1, without explanatory variables). As shown in Model 2, the presence of movement has a significant influence on the count level. The presence of movement yielded significantly higher counts than the absence of movement with a count difference of 29.1 between the presence versus absence of upper body movement. In addition, including a random slope at level‐3 for the variable occurrence of movement significantly improves model fit, χ^2^ = 84.7 (1), *p* < .05 showing that the relationship between the occurrence of movement and count level significantly differs between participants (Model 3). Based on Model 3, on average, the presence of movement resulted in 2.3 times higher counts compared with the absence of movement (count difference of 28.7 between the presence versus absence of upper body movement). Thus, the presence versus absence of movement significantly increased the count level. The residuals of the final model were, however, not normally distributed. Therefore, as a sensitivity analysis, a logistic model for binomial responses was constructed. The logit model confirmed our findings of the linear model and did show a 4.5 times higher risk (measured in odds) of higher category counts for the presence of movement versus absence of movement (see Table 3). This suggests that the Actiwatch‐2 is able to detect changes in the occurrence of upper body movement.

**Table 2:** The mean activity counts and standard deviations for the absence and presence of movement among different activity situations and activities performed from different body positions

Occurrence of movement	Mean	*SD*	*N*
Absence of movement	7.2	29.0	4,558
Body position			
Lying	2.7	12.5	1,484
Sitting	9.4	34.2	3,031
Standing still	5.6	11.4	7
Standing/moving	11.2	14.8	36
Activity situation			
Being present	5.5	20.0	3,542
Being part of	18.6	58.6	655
Passive participation	3.5	9.0	193
Active participation	5.0	14.8	168
Presence of movement	90.1	139.3	3,685
Body position			
Lying	105.2	158.7	923
Sitting	85.3	132.4	2,736
Standing still	109.7	52.7	9
Standing/moving	35.2	44.3	17
Activity situation			
Being present	44.8	72.8	1,750
Being part of	85.7	118.8	515
Passive participation	60.4	66.0	301
Active participation	171.0	195.0	1,119

Abbreviations: *SD* = standard deviation, *N* = total number of valid observations with simultaneous activity counts for the different categories.

**Table 3:** Multilevel models to explain the activity counts

	Model 1	Model 2	Model 3	Model 4	Model 5	Logit model	
	*b* (SE)	*b* (SE)	*b* (SE)	*b* (SE)	*b* (SE)	*b* (SE)	e^b^
Intercept	38.68 (11.06)	26.07 (9.93)	21.89 (7.43)	15.75 (10.00)	16.58 (7.08)	−1.55 (0.24)	0.21
Occurrence of movement (Present)		29.12 (1.98)*	28.65 (5.21)*	28,72 (5.21)*	26.42 (5.22)*	1.50 (0.13)*	4.48
Body position (Sitting)				8.65 (9.51)			
Body position (Standing still)				−43.91 (17.77)*			
Body position (Standing/moving)				−5.43 (14.07)			
Activity situation (Being part of)					16.43 (3.00)*	0.49 (0.10)*	1.63
Activity situation (Passive participation)					22,80 (5.66)*	1.03 (0.17)*	2.80
Activity situation (Active participation)					16.41 (4.29)*	0.30 (0.13)*	1.35
Level 3 variance	1,850.17 (807.67)	1,374.86 (643.57)					
Intercept			532.34 (364.51)	494.49 (353.35)	457.09 (326.16)	1.03 (0.36)	
Slope			478.19 (177.54)	477.45 (177.10)	474.99 (176.12)	0.21 (0.10)	
Level 2 variance	3,792.27 (555.67)	3,548.77 (520.39)	3,314.31 (482.79)	3,361.86 (489.03)	3,111.51 (452.85)	0.45 (0.08)	
Level 1 variance	4,511.17 (70.75)	4,404.03 (69.10)	4,350.86 (68.35)	4,346.92 (68.29)	4,333.52 (68.08)		
−2Loglikelihood	93,337.41	93,052.13	92,967.45	92,960.41	92,925.90		

* Estimated coefficient is larger than two times its standard error. *b* = coefficient estimates. e^b^ = odds ratios.

### 3.3 Relationship with body position and activity situation

Table 3 shows the influence of body position (Model 4) and activity situation (Model 5) on the count level. Sitting and standing/moving versus lying had no significant influence on the count level, while standing still yielded significantly lower counts than lying. However, standing still is based on only four minutes of observation (seven and nine video‐based observations related to the absence and presence of movement, respectively (see Table 2)), which made this result unsuitable for interpretation. Moreover, including the body position did not significantly improve the model fit, χ^2^ = 7.0 (3), *p *> .05. This is confirmed by the logit model showing no relationship with body position.

When adding activity situation to model 3, being part of, passive participation, and active participation yielded significantly higher counts than being present. When using being part of, passive participation or active participation as reference variable, a significant effect of being present only was found. Including the activity situation significantly improved the model fit, χ^2^ = 41.6 (3), *p* < .05 (Model 5 vs. Model 3). Based on the final model (Model 5), the mean number of counts of all participants was 16.6. With the presence of movement, the count level improved with 26.4 counts. In addition, involvement in the activity situation leads to an improvement in counts (being part of: 16.4, passive participation: 22.8, active participation: 16.4) in comparison with a situation in which people with PIMD were not involved in the activity (being present). The logit model confirmed a relationship between activity situation and counts (see Table 3). The probability of higher category counts increased with involvement in an activity situation. The risk (in terms of odds) of higher category counts for being part, passive participation and active participation were 1.6, 2.8, and 1.4 times the risk of being present, respectively (Logit model, Table 3).

In summary, the Actiwatch gives significantly higher counts for the presence versus absence of movement and significantly higher counts for three of the activity situations versus being present when adding activity situation in addition to the occurrence of movement (Model 5 and Logit model). There is, however, a large variance between participants when it comes to the counts that are associated with the occurrence of movement and activity situation. The level‐3 variance (between participants) of the intercept and slope is 457.1 and 475.0 (Model 5, Table 3).

## 4 DISCUSSION

### 4.1 Main findings

This study investigated the construct validity of the Actiwatch‐2 to assess the occurrence of upper body movement in people with PIMD. The major finding is that the Actiwatch‐2 is able to distinguish the presence of upper body movement from the absence of upper body movement in people with PIMD. This study did not find a significant effect of body position, in particular of a lying and sitting position, on the count level, In addition, the Actiwatch‐2 gave significantly higher counts in situations in which a person with PIMD is involved in the activity from situations in which a person with PIMD is present but not involved. The Actiwatch‐2 is, however, not able to distinct different types of activity at which people with PIMD are involved. For instance, the Actiwatch is not able to distinguish if the presence of movement is derived from massage (being part of), swinging in a hammock (passive participation) or initiated bouncing movements (active participation). In addition, the results showed a wide variety in the count range between participants. Therefore, cut‐off values should be defined person‐to‐person.

### 4.2 Theoretical reflection and implications

The validity evidence with regard to the measurement of physical activity in people with intellectual disability, and in particular in people with PIMD, is limited (Pitchford et al., 2018). This study provides evidence with regard to the construct validity of the Actiwatch‐2 as a measurement of movement in people with PIMD. The results can be used to evaluate interventions directed at the facilitation of upper body movement of people with PIMD. The Actiwatch‐2 may be suitable to determine whether movement activity results from facilitation and whether obvious movements instead of none or small involuntary movements (scored as the absence of movement) were seen in people with PIMD. In general, an Actiwatch is sensitive for small movements and will register involuntary movements (Warms, 2006). However, based on the current study and those of Warms and Belza (2004) it can be suggested that significant movement activity resulting from facilitation can be distinguished from small involuntary movements based on the individual count pattern. This finding is important, given that small involuntary movements due to spasticity, epilepsy and stereotypical behaviour are common in people with PIMD (Poppes, Van der Putten, & Vlaskamp, 2010; Van Timmeren, Van der Putten, Van Schrojenstein Lantman‐de Valk, Van der Schans, & Waninge, 2016; Van der Heide, Van der Putten, Van den Berg, Taxis, & Vlaskamp, 2009). In addition, with the ability of the Actiwatch‐2 to distinguish the presence and absence of movement, the Actiwatch‐2 may be useful to evaluate whether inactivity has decreased in people with PIMD. This is important, because these people are at risk for being physically inactive (Bjornson, Belza, Kartin, Logsdon, & McMaughlin, 2007; Hilgenkamp et al., 2012; Van der Putten et al., 2017) and even small improvements in physical activity can be very beneficial for these persons (Jones et al., 2007; Levine, 2007; Woodcock, Franco, Orsini, & Roberts, 2011).

Based on the results with regard to activity situation, the outcomes of the Actiwatch‐2 can be best explained with both the occurrence of movement and activity situation added to the model. In addition, the Actiwatch‐2 give significantly higher counts in situations in which a person with PIMD is involved in the activity in comparison with situations in which a person with PIMD is present but not involved. Therefore, we suggest that activities including the stimulation of social interaction, tactile stimulation or stimulation of the motor domain could possibly be distinguished from none and audio‐visual activities, such as watching television or listening to music. This finding may contribute to future research emerged at improvement of the quality of support of people with PIMD (Van der Putten & Vlaskamp, 2011). The current study, however, showed, that the Actiwatch is not able to distinct activity situations with the involvement of a person with PIMD and thus between movement activities and activities such as massage. An explanation might be that the functional use of the arms of people with PIMD is limited (Nakken & Vlaskamp, 2007) resulting in movements with a low frequency, intensity and duration independent of the type of stimulation. Based on the variety in count range for the occurrence of movement between participants, it is possible that the ability in handling objects differed between participants. A less severe limitation in motor functioning may increase the active participation including the speed of performed movements and therefore may increase the accuracy of measurement in people with PIMD. For future studies, it is, therefore, recommended that an individual approach is used or that the manual ability of the participants is included as factor in the analysis.

With regard to the relationship between body position and counts, the Actiwatch did show similar outcomes for different body postures and is, similar to the ability of accelerometers in general, limited in accurately measuring body postures (Ainsworth et al., 2015). One explanation might be the place where the Actiwatch is worn. An accelerometer device worn on the leg has been shown to accurately measure reductions in sitting time (Kozey‐Keadle et al., 2011). It may be that just wrist derived data, as collected in the current study, are inappropriate to distinct between body postures.

**Figure 2:**
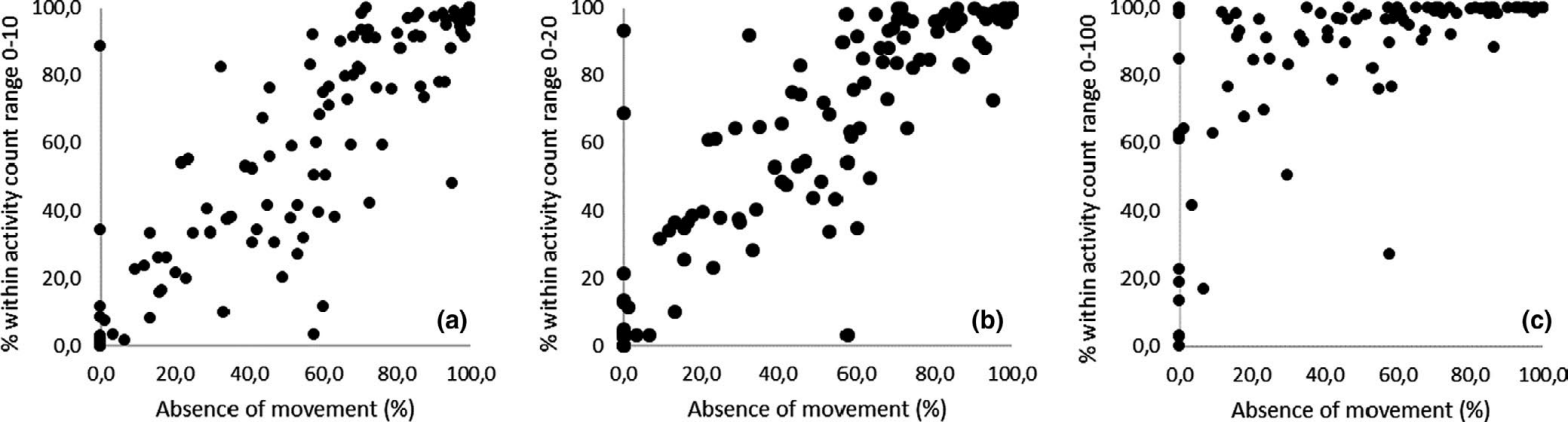
Percentage observed as the absence of movement related to the percentage scored within count range: 0–10 (a), 0–20 (b), and 0–100 (c)

This study concludes a difference in counts for the absence versus presence of upper body movement as well as for an activity situation in which participants were involved versus not involved. Nevertheless, thresholds to summarize the counts into specific activity categories for persons with PIMD have to be further calibrated. This is important in order to be able to predict the time spent within different movement activity and evaluate the effect of interventions aimed at the facilitation of movement or reduction of inactivity in people with PIMD. The output of accelerometers is usually summarized into categories such as sedentary, light, moderate and vigorous‐intensity activity expressed in terms of energy expenditure or metabolic equivalents (METs) to gain further insight in the physical activity patterns (Ainsworth et al., 2015). People with PIMD are, however, dependent on substantial assistance and perform mostly non‐ambulatory activities (Van der Putten et al., 2017). In addition, there is a lack of algorithms that predict the energy expenditure of people with PIMD (Strath, Pfeiffer, & Whitt‐Glover, 2012; Waninge et al., 2013), making the intensity of their activities unclear. For each group and type of activity (for all types of inertial measurement units including accelerometer data), data filters as well as specific algorithms are needed in order to make the data usable and understandable. To determine the performed movement of persons with PIMD in practice, it is important to consider PIMD‐specific categories (such as we did in current study by defining activity situations) to summarize the data. In addition, it should be taken in mind that prediction equations should also be validated (Plasqui & Westerterp, 2007). Moreover, as the group of people with PIMD is heterogeneous (Nakken & Vlaskamp, 2007) and the relationship between the occurrence of movement and outcomes of the Actiwatch significantly differs between participants, cut‐off values should be individually defined. To be able to predict the outcomes of the Actiwatch‐2 that relate to activity and inactivity for an individual with PIMD, an individual count pattern could be investigated by the use of video observation in combination with Actiwatch‐2 measurements. Although group‐based comparative intervention‐based research in people with PIMD seems inappropriate, we want to emphasize with a rough indication for inactivity that the outcomes for this target group should be viewed differently. A count range 0–100 (or even upto 145 counts: Neil‐Sztramko et al. (2017)) is usually seen as low category counts (Van Alphen et al., 2016). However, when analysing the effects of interventions on group level, a first and rough indication can be obtained by using a count range between 0 and 15 for inactivity (i.e. the absence of movement) in people with PIMD. This recommendation is based on the ceiling effect shown in Figure 2 and the intercept of Model 5, Table 3, which should be fine‐tuned and tailored on an individual level.

### 4.3 Methodological reflection and implications

When interpreting the results of this study a few remarks need to bear in mind. As this study was a validity study, outliers were not excluded from the analysis. As stated in the result section, the mean number of counts for all participants except participant 3 was higher for the presence versus absence of movement. The data of this participant seem to be influenced by one video recording presenting a mean count of 218.8 while no actual performance of movement was observed. Based on the count pattern, it seems that the Actiwatch had been stuck in the value during the performance of movement earlier in the video. Although further analysis showed that exclusion of this video remains the same study conclusion, it needs to be taken in mind that such deviations may occur by using technical devices such as an Actiwatch. Despite that, it has also been showed that the correlation between measured and true exposure was higher for accelerometers compared with questionnaire measurements (Ferrari, Friedenreich, & Matthews, 2007). However, it should be taken in mind that the Actiwatch may not suitable for every person with PIMD. In the current study, 11.5% of the participants had to be excluded due to oversensitivity or reluctance to wear the device on their wrist.

The data used in current study were retrieved from three measurement periods at which Actiwatch and video data were collected. As we could only use the data from similar periods of time and based on valid conversion of the timeframes of the camera to the Actiwatch, not all participants had data in all categories with regard to upper body movement, body position and activity situation. Data have not been used in the absence of a first frame with a sheet indicating the time of the day for validation of the time frames. In addition, the manually set time of one of the cameras used was untraceable for the time of measurement and related data could therefore not be included. Data had to be excluded when necessary and are therefore randomly determined per person and category. Despite this random allocation, the exclusion of data is a limitation with regard to the total of valid observations as well as the distribution of data in each of the categories. For future research, a primary focus on the study purpose is recommended to have an equal distribution of data in each of the categories. In addition, although persons with GMFCS IV are able to walk with physical assistance, unsurprisingly, standing and walking/moving were seen very infrequent in the current study. Therefore, the influence of body position on the outcomes of the Actiwatch‐2 should be further investigated in persons with PIMD.

The distribution of counts in the current study was right skewed. Therefore, as a sensitivity analysis, a logistic model for binomial responses was constructed. The multilevel and logistic model used reveals the same conclusion about the capabilities of the Actiwatch‐2 with regard to the assessment of movement, body position, and activity situation. In addition, a logistic model containing a count range 0–100 as low category counts (which is usually seen as sedentary time although based on different Actigraph devices; Lambiase et al., 2014; Van Alphen et al., 2016) also remains the same conclusion. However, a count range containing 0–100 for inactivity does not apply to people with PIMD (See Figure 2). Further research with an individual approach is required to predict the outcomes of the Actiwatch‐2 based on observations of upper body movement, body position and activity situation for a person with PIMD. Therefore, caution is needed with prediction modelling based on current results.

In the general population, a sitting and lying position with or without movement are seen as sedentary behaviour which is detrimental to health (Kozey‐Keadle et al., 2011; Owen, Healy, Matthews, & Dunstan, 2010). In the current study, however, we mindfully included the data collected in a lying and sitting position because even facilitation of small movements of the limbs might be important for people with PIMD to increase their active participation and interaction within daily activities. We excluded, however, the very small sliding hand movements and minimal vibrating movements for reliability reasons regarding coding and because those are usually not proposed by movement interventions. Despite that, movements that are almost invisible by the eye, such as subtle head movements, could be of interest for instance in stimulating effective interaction. We suggest that in case of subtle head movements, for instance, techniques such as motion history (Iwabuchi et al., 2014) or simplified motion energy analysis (Van Delden & Reidsma, 2018) can be considered better instead of an Actiwatch that is based on wrist movements. Future research should, therefore, be clear in their (intervention) purpose to determine what type of movements should be included when studying people with PIMD. In addition, as passive movements of the whole body, such as being moved in a hammock, do not rely on an active involvement with trunk and arm movements of people with PIMD, these passive movements of the whole body are not scored in the current study as the performance of movement. This may be a limitation with regard to the assessment of movement of people with PIMD. However, it can actually be discussed if those kind of movements should be seen as movement when there is no active participation of the person with PIMD. This type of activity includes sensory stimulation (e.g. experiencing the wind while swinging) as well as vestibular stimulation which may evoke reflex responses in the muscles (Mittal & Narkeesh, 2012). As these type of activities are used in current practice to activate people with PIMD (Van Alphen et al., 2019) and may evoke a motor response, professional consensus about what movement should actually consist of for people with PIMD is needed. In addition, this study did not identify movements of the legs. Although this can be seen as a limitation, we expect other instruments than the Actiwatch to be needed to identify leg movements. Most movements of people with PIMD are performed from a lying and sitting position and we do not expect leg movements from these body postures to influence wrist movements. An accelerometer device worn on the leg could possibly offer a solution here (Kozey‐Keadle et al., 2011).

Based on the current study, it can be suggested that the Actiwatch‐2 is able to distinguish obvious movement activity from small involuntary movements. Spasticity and stereotypical behaviour, however, could also manifest as obvious limb movements, identified as the presence of movement. Although these movements are a form of activity (Warms & Belza, 2004), those are usually not aimed to improve by interventions (although they could be in some cases an expression of enthusiasm). Moreover, to our opinion, frequently seen stereotypical behaviour hamper the opportunity to explore the environment and the development of functional skills. However, identifying those involuntary movements with an Actiwatch is difficult, because the acceleration signal related to movement needed for manipulating material could in fact be the same as the acceleration signal related to movement shown during stereotypical behaviour. Therefore, for future research, it is recommended that participants with significant involuntary movements be analysed separately in a way that the movement elicited can be distinguished from involuntary movements. In addition, it is important to maximize the benefits of movement in persons with PIMD by integrating individual tailored movement activities into their support.

## 5 CONCLUSION

The Actiwatch‐2 may be useful to assess the occurrence of movement of people with PIMD, and whether there is involvement in an activity situation. However, further studies are needed to calibrate cut‐off points to define the counts and patterns of change for individuals with PIMD.

## CONFLICT OF INTEREST

There are no conflicts of interest.
